# Effect of electroacupuncture combined with sulforaphane in the treatment of sarcopenia in SAMP8 mice

**DOI:** 10.22038/IJBMS.2024.71345.15509

**Published:** 2024

**Authors:** Fei Guo, Linlin Fu, Zhenchan Lu

**Affiliations:** 1 Department of TCM Acupuncture, Huzhou Central Hospital & Affiliated Central Hospital Huzhou University, 313000, Huzhou, China; 2 Department of Pathology, Huzhou Central Hospital & Affiliated Central Hospital Huzhou University, 313000, Huzhou, China; 3 Department of Neurology, Huzhou Central Hospital & Affiliated Central Hospital Huzhou University, 313000, Huzhou, China

**Keywords:** Anti-oxidants, Electroacupuncture, Mitochondria, Sarcopenia, Sulforaphane

## Abstract

**Objective(s):**

Skeletal muscles mitochondrial dysfunction is the main cause of sarcopenia. Both electroacupuncture (EA) and sulforaphane (SFN) have been shown to improve oxidative stress and inflammation levels to maintain mitochondrial function, but the effects and mechanisms of their combination on sarcopenia are unclear. This study aimed to investigate the regulatory effects of EA combined with SFN on sarcopenia.

**Materials and Methods:**

SAMP8 mice were used and intervened with EA or SFN, respectively, and Masson and HE staining were used to observe pathological changes in skeletal muscle tissue. Transmission electron microscopy was used to detect tissue mitochondrial changes. TUNEL staining was used to assess apoptosis. The biochemical and molecular content was tested by ELISA, western blot, and qRT-PCR.

**Results:**

The results showed that oxidative stress, apoptosis, and IL-6, TNF-α, Atrogin-1, and MuRF1 levels in skeletal muscles cells were suppressed and mitochondrial damage was repaired after EA or SFN intervention. In addition, we found that the above changes were associated with the activation of the AMPK/Sirt1/PGC-1α pathway in skeletal muscle tissues, and the promotion effect of combined EA and SFN intervention was more significant.

**Conclusion:**

In conclusion, this study found that EA combined with SFN mediated the repair of mitochondrial damage through activation of the AMPK/Sirt1/PGC-1α pathway, thereby alleviating skeletal muscles morphology and function in sarcopenia. This study combines EA with SFN, which not only broadens the use of electroacupuncture and SFN but also provides a scientific experimental basis for the treatment of sarcopenia.

## Introduction

Sarcopenia, first named by Rosenberg, presents as an age-related loss of muscle mass and muscle strength (1). It is associated with the occurrence of adverse events such as impaired mobility, falls, fractures, and physical disability in older people, and places a great burden on families and society (2). Age-related aging of skeletal muscles and mitochondrial dysfunction are the main causes of sarcopenia (3), making the maintenance of mitochondrial homeostasis a high priority in the treatment of sarcopenia.

Mitochondria are the main site of energy synthesis in skeletal muscles cells and their number is critical to skeletal muscles function (4). In addition, the dynamic balance between reactive oxygen species and anti-oxidants is closely related to the functional homeostasis of mitochondria, which provide energy while generating large amounts of reactive oxygen species (5). The AMPK/Sirt1/PGC-1α signaling pathway can promote mitochondrial biogenesis and improve mitochondrial function (6). Studies have shown that the PGC-1α signaling cascade can activate nuclear factor E2-related factor 2 (Nrf2) and improve oxidative stress to maintain muscle energy metabolism (7). Nrf2 is an anti-oxidant damage transcription factor that regulates the production of downstream anti-oxidant proteins such as catalase (CAT) and superoxide dismutase (SOD). Skeletal muscles senescence promoted by Nrf2 deficiency is a potential mechanism for the development of skeletal sarcopenia (8). Therefore, Nrf2 is expected to be a new target for the prevention and treatment of sarcopenia.

Sulforaphane (SFN), the Nrf2 signaling pathway activator, is a naturally occurring dietary isothiocyanate (ITC) produced by the enzymatic transformation of mustard oleoresin by black mustard enzymes (9). Recent studies have shown that SFN inhibits inflammation and apoptosis by up-regulating the Sirt1/PGC-1α/Nrf2 pathway in cells (10). SFN can also attenuate oxidative stress levels in human granulosa lutein cells by activating AMPK and NRF2 (11). Another study confirmed that SFN prevents age-related cardiac and muscle dysfunction through the Nrf2 signaling pathway (12). Therefore, we hypothesize that SFN may reduce mitochondrial oxidative stress levels and improve skeletal muscles function in sarcopenia through activation of the AMPK/Sirt1/PGC-1α/Nrf2 signaling pathway.

Electroacupuncture (EA) is the use of low-frequency electrical currents to excite acupuncture tissues, providing both acupuncture and electrical stimulation (13). EA has been found to delay muscle atrophy in aging mice by modulating the pro-angiogenic process and protein turnover in the gastrocnemius muscle (14). Furthermore, EA targets the expression of PGC-1α and p-AMPK in the skeletal muscles of diet-induced obese rats, suggesting its efficacy in restoring fatty acid oxidation and improving mitochondrial function in skeletal muscles cells (15). However, EA combined with SFN in sarcopenia is yet to be investigated.

Therefore, the present study was designed to investigate the effects of SFN and EA on the improvement of skeletal muscles function and molecular mechanisms in mice with sarcopenia by observing the morphological changes, oxidative stress levels, expression of AMPK/SIRT1/PGC-1ɑ pathway and downstream proteins in the skeletal muscles of SAMP8 mice. This study is expected to provide a scientific basis for clinical intervention in sarcopenia and the combined application of SFN and EA.

## Materials and Methods

***Animal ethics***

Three-month-old adult male senescence-accelerated mice (SAMP8), weighing 35 g, were purchased from Hangzhou Hangsi Biotechnology Co., Ltd, SCXK (jing) 2020-0004. The Animal Experimentation Ethics Committee of Zhejiang Eyong Pharmaceutical Research and Development Center is responsible for ethical review, SYXK (Zhejiang) 2021-0033.

***Reagent information***

SFN was purchased from Shanghai Yuanye Co., Ltd. and is soluble in water and DMSO. Masson and HE staining solutions were purchased from Servicebio. TUNEL kit, proteinase K, DAPI, and PBS were purchased from Servicebio. BCA protein quantification kits were obtained from Solarbio. SOD1 and SOD2 kits were purchased from Shanghai Enzyme Link. Ltd. The GPX1 kits were obtained from Jiangsu Enzyme Immunity Industry Co. PXDN and CAT kits were purchased from Beijing Donggeboye and Nanjing Jiancheng Co.

***Group setup and sample collection***

Twenty-four mice were randomly divided into 4 groups of 6 mice each: SAMP8 group, SAMP8+EA group, SAMP8+SFN, SAMP8+EA+SFN. The SFN intervention group was treated at 4 mg/kg gavage twice a week for 4 weeks (13). EA treatment was carried out according to the reported protocol after fixating mice with homemade mouse fixators (16**)**. Selected acupuncture points of Guanyuan (CV 4), Zusanli (ST 36), and Baihui (DU 20) were used. The mice were treated with 0.20×13 mm disposable milli-needles and EA (Korean type LH202H). The mice were treated with continuous waves at a frequency of 2 Hz and an intensity of 1 mA. The mice were acupunctured once a day and treated 3 times a week for 15 min each time with EA for a total duration of 4 weeks. Then skeletal muscle tissue and peripheral blood were collected and preserved.

***Masson staining***

Paraffin sections of skeletal muscle tissue were dewaxed in xylene I and xylene II solutions and ethanol. The subsequent operations were completed according to the instructions of the Masson staining kit. The results are interpreted based on: blue color for collagen fibers; and red color for myofibres, fibrin, and red blood cells.

***ELISA***

The activities of CAT, SOD1, SOD2, PXDN, and GPX1 in tissues were assayed by the kits as described above. Skeletal muscle tissue was washed in physiological saline and blotted on filter paper, then 100 mg of tissue was cut and made into a 10% homogenate. Reagents, samples, and standards were prepared according to the kit requirements. The enzyme reagent and color development solution were added, and the termination solution was added after sufficient reaction. The OD values were measured to assess the activity.

***HE staining***

HE staining was used to observe the morphology of skeletal muscle tissue in various groups of mice. Skeletal muscle tissues were dewaxed and stained with hematoxylin and eosin solution to observe the tissue morphology under a microscope.

***TUNEL staining***

TUNEL staining was used to observe the apoptosis of mouse skeletal muscle tissue. The kit (Servicebio, G1501) was FITC fluorescein-labeled and the nuclei of positive apoptotic cells were green. Normal cell nuclei were stained blue by DAPI.

***Reverse transcription and quantitative PCR (qRT-PCR)***

One thousand microliters of Trizol were added to each 200 mg of skeletal muscle tissue, and mRNA was fully lysed. mRNA was purified by repeated centrifugal extraction. Reverse transcription reactions were carried out at 42 °C for 15 min and 85 °C for 5 min. Real-time fluorescent quantitative PCR reactions were then performed according to the operating instructions. Primer sequences of PCR information are shown in Table 1.

***Transmission electron microscopy***

Before transmission electron microscopy observation, skeletal muscle tissue had to be fixed in 2.5% glutaraldehyde solution and 1% osmium solution, respectively. Samples were dehydrated, embedded, and polymerized before being stained with UO_2_ acetate and lead citrate solutions. The samples were observed by electron microscopy after the above treatments.

***Western blot***

100 mg of mouse skeletal muscle tissue was prepared as a tissue homogenate. After full lysis, the tissue was centrifuged at 12000 g for 5 min at 4 °C and then the concentration was determined by the BCA kit. SDS-PAGE was carried out according to the instructions to obtain the target bands. After membrane transfer and antigen-antibody reaction, ECL chemiluminescence was used to visualize the protein bands. Image J software was used to detect the grayscale values of strips for semi-quantitative analysis. Antibody information is shown in Table 2.

***Statistical analysis***

For data analysis, SPSS 16 statistical software was utilized, and all data were represented as mean±standard deviation. One-way ANOVA analysis was performed for multi-group measurements that were normally distributed, and Chi-square and Tukey’s test was utilized for two-way comparisons across groups. Dunnett’s T3 test or independent samples t-test was used when the data were normally distributed but had uneven variances; Otherwise, the Kruskal-Wallis H-test was used. *P*<0.05 was considered a statistically significant difference.

## Results

***EA combined with SFN inhibits interstitial fibrosis in the skeletal muscles of SAMP8 mice***

**Figure 1:**
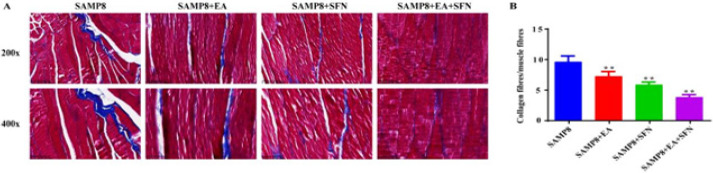
EA combined with SFN inhibits skeletal muscle interstitial fibrosis in SAMP8 mice

The muscle tissue of SAMP8 mice was observed after Masson staining, the collagen fibers in the tissue were stained blue and the muscle tissue was stained red (Figure 1A). The muscle tissue of SAMP8 group mice had an uneven distribution of myofibres and collagen fibers, a large number of collagen fibers exuded, the interstitial space of myofibres increased, and the overall structure and arrangement was disordered (Figure 1A). In the SAMP8+EA, SAMP8+SFN, and SAMP8+EA+SFN groups, the distribution of collagen fibers and smooth muscle fibers in the muscle tissue was more even, and the muscle fibers were normal, full, neatly arranged, and dense (Figure 1A). The above changes were most significant in the SAMP8+EA+SFN group (Figure 1A). We found that the collagen fiber/myofibre ratio was significantly lower in the skeletal muscle tissue of mice in the intervention group compared to the SAMP8 group (Figure 1B).

***EA combined with SFN promotes anti-oxidant protein expression and inhibits oxidative stress in SAMP8 mice***

**Figure 2:**
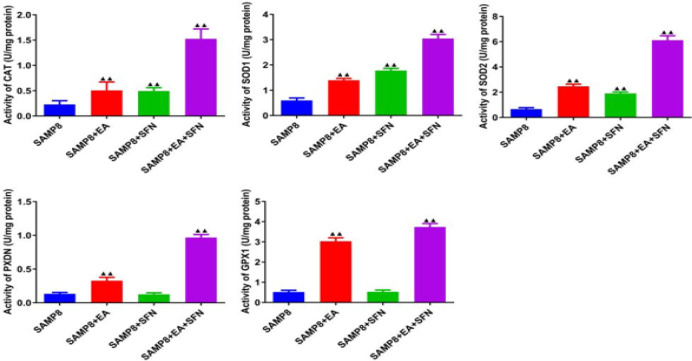
EA combined with SFN promotes anti-oxidant protein expression and inhibits oxidative stress in SAMP8 mice

ELISA was used to detect changes in the levels of CAT, SOD1, SOD2, PXDN, and GPX1 in mice (Figure 2). Compared with the SMAP8 group, the levels of CAT, SOD1, SOD2, PXDN, and GPX1 were significantly higher in the SAMP8+EA and SAMP8+EA+SFN groups (Figure 2). The CAT, SOD1, and SOD2 levels were significantly higher in the SAMP8+SFN group, while PXDN and GPX1 were not significantly different (Figure 2).

***EA combined with SFN promotes the recovery of skeletal muscle tissue in SAMP8 mice to inhibit apoptosis***

**Figure 3:**
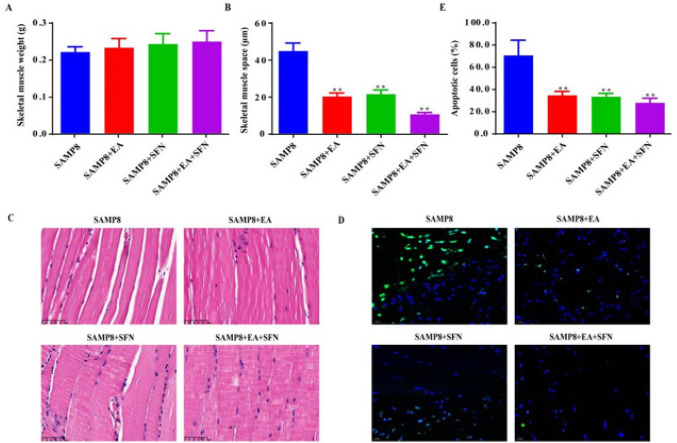
EA combined with SFN promoted the recovery of skeletal muscle tissue weight and morphology and inhibited skeletal muscle cell apoptosis in SAMP8 mice

Compared with the SAMP8 group, skeletal muscle weight was not significantly different between the SAMP8+EA and SAMP8+EA+SFN groups, but skeletal muscle space was significantly lower (Figure 3A-3B). HE staining was used to observe the morphology of the skeletal muscles in each group (Figure 3C). The results indicated that there were a large number of broken and disintegrated skeletal muscle fibers in the SAMP8 group, infiltrated by inflammatory cells and disorganized myofibrils (Figure 3C). In the SAMP8+EA and SAMP8+SFN groups, there were broken and fragmented myofibres but the pathology was significantly improved and the myofibres were fuller compared to the SAMP8 group (Figure 3C). In the SAMP8+EA+SFN group, the skeletal muscle myofibrils were structurally intact, the myofilaments were neatly arranged, the myocytes were normal, and a few inflammatory cells were seen (Figure 3C). Apoptosis of mouse skeletal muscle tissue cells was detected by TUNEL staining (Figure 3D-3E). The rate of positive cells in skeletal muscle tissue was significantly reduced in all other groups compared to the SAMP8 group (Figure 3D-3E).

***EA combined with SFN reduces muscle tissue inflammatory factor levels and inhibits skeletal muscle degradation***

**Figure 4:**
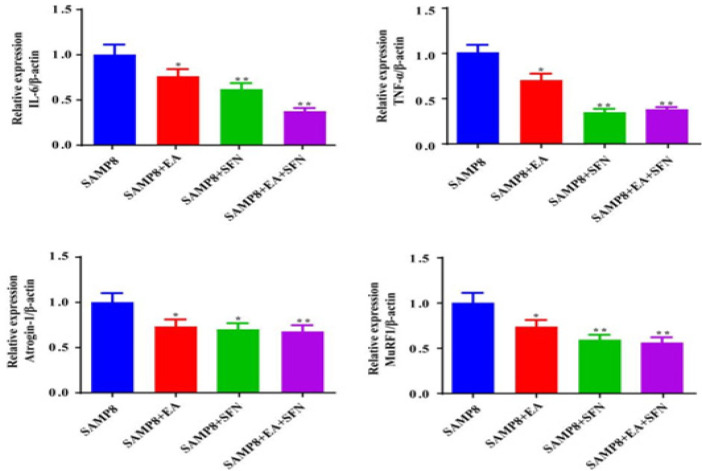
qRT-PCR was used to detect IL-6, TNF-α, Atrogin-1, and MuRF1 mRNA expression in mouse muscle tissues

qRT-PCR was used to detect IL-6, TNF-α, Atrogin-1, and MuRF1 mRNA levels in mouse muscle tissues (Figure 4). The results suggested that the expression of IL-6 and TNF-α was suppressed and the expression levels of Atrogin-1 and MuRF1 were reduced in mouse muscle tissues after the combined intervention of EA, SFN, EA, and SFN compared with the SAMP8 group (Figure 4).

***EA combined with SFN increases the number of mitochondria in the skeletal muscle tissue of SAMP8 mice***

**Figure 5:**
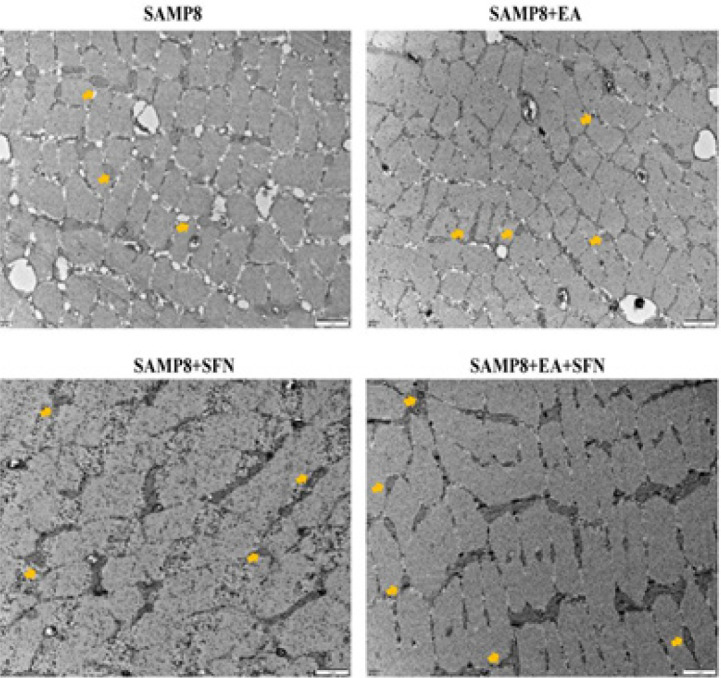
EA combined with SFN restores mitochondrial morphology and increases their number in skeletal muscle tissue of SAMP8 mice

Transmission electron microscopy was used to observe mitochondrial changes in muscle tissue (Figure 5). In the SAMP8 group, the myogenic fibers were blurred, and arranged in a disorganized manner, with unclear transverse lines and deformed and ruptured mitochondria, while in the SAMP8+EA, SAMP8+SFN, and SAMP8+EA+SFN groups, pathological damage to the muscle tissue was alleviated and the number of mitochondria increased compared to the SAMP8 group (Figure 5). In the SAMP8+EA+SFN group, the myogenic fibers were clearer and neater, and the mitochondria were arranged in beads in the fibers, with a more normal size and shape (Figure 5).

***EA combined with SFN increases AMPK/Sirt1/PGC-1α pathway protein expression in skeletal muscle tissues of SAMP8 mice***

**Figure 6:**
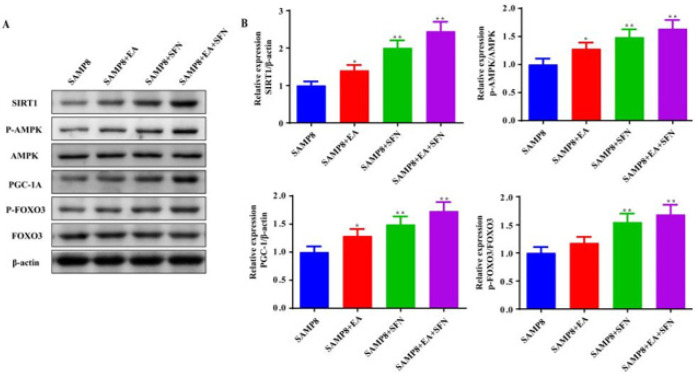
EA combined with SFN increases AMPK/Sirt1/PGC-1α pathway protein expression in skeletal muscle tissues of SAMP8 mice

Western blot was used to detect pathway protein expression levels in muscle tissue (Figure 6A-6B). The expression levels of SIRT1, p-AMPK, and PGC-1α were significantly higher in the skeletal muscles of SAMP8+EA mice compared with the SAMP8 group (Figure 6A-6B). Compared to the SAMP8 group, SIRT1, P-AMPK, PGC-1α, and P-FOXO3 protein expression levels were significantly increased in skeletal muscles of SAMP8+SFN and SAMP8+EA+SFN groups (Figure 6A-6B).

**Table 1:** Primer sequences of the genes detected by qRT-PCR in SAMP8 mice

Gene	Forward Primer (5’ to 3’)	Reverse Primer (5’ to 3’)
Mouse TNF-α		
Mouse IL-6	TCCGGAGAGGAGACTTCACA	CATAACGCACTAGGTTTGCCG
Mouse Atrogin-1		
Mouse MuRF1	CCAGGCTGCGAATCCCTAC	ATTTTCTCGTCTTCGTGTTCCTT
Mouse β-actin	GTGACGTTGACATCCGTAAAGA	GCCGGACTCATCGTACTCC

**Table 2:** Antibody information of the proteins detected by Western blot in SAMP8 mice

Antibody name	Company		Number	Dilution ratio
SirT1 Antibody	Affinity		DF6033	1: 1000
Phospho-AMPK alpha (Thr172) Antibody	Affinity		AF3423	1: 1000
AMPK alpha Antibody	Affinity		AF6423	1: 1000
PGC1 alpha Antibody	Affinity		AF5395	1: 1000
Phospho-FOXO3A (Ser413) Antibody	Affinity		AF2343	1: 1000
FOXO3A Antibody	Affinity		AF7624	1: 1000
β-actin Antibody	Affinity	AF7018		1: 10000

(A) Masson staining was used to detect the degree of skeletal muscle fibrosis in SAMP8 mice (magnification ×200, 400). (B) EA combined with SFN reduced the collagen fiber/ muscle fiber ratio in SAMP8 mice. EA: Electroacupuncture; SFN: Sulforaphane

n=6, *P<0.05, **P<0.01 vs SAMP8 group. EA: Electroacupuncture; SFN: Sulforaphane

(A-B) Skeletal muscle tissue weight and space were examined in each group of SAMP8 mice.

(C) Skeletal muscles histopathological changes in mice by HE staining (magnification, ×400).

(D-E) TUNEL staining was used to detect apoptosis in mouse skeletal muscle tissue (magnification, ×400). n=6, *P<0.05, **P<0.01 vs SAMP8 group.

EA: Electroacupuncture; SFN: Sulforaphane

n=3, *P<0.05, **P<0.01 vs SAMP8 group.

EA: Electroacupuncture; SFN: Sulforaphane

n=6, *P<0.05, **P<0.01 vs SAMP8 group.

EA: Electroacupuncture; SFN: Sulforaphane

(A-B) Detection of SIRT1, p-AMPK, PGC-1α, and p-FOXO3 protein expression in skeletal muscles of mice by western blot. n=3, *P<0.05, **P<0.01 vs SAMP8 group.

EA: Electroacupuncture; SFN: Sulforaphane

## Discussion

In this study, we demonstrated that EA combined with SFN had synergistic effects, which were effective in alleviating sarcopenia in SAMP8 mice. We found that EA combined with SFN restored skeletal muscle function in sarcopenia by activating the AMPK/Sirt1/PGC-1α pathway, inhibiting oxidative stress, apoptosis, and inflammation levels in skeletal muscle cells, and increasing the number and function of skeletal muscle mitochondria.

The use of SAMP8 mice as a model of sarcopenia has been widely reported (17). Disruption of skeletal muscle structure and imbalance of fiber ratio are typical symptoms of sarcopenia (18). In the present study, we also found skeletal muscle fiber breaks, collagen fiber leaks, and an enlarged muscle fiber gap in SAMP8 mice. Sarcopenia is associated with aging, with persistent inflammation and oxidative stress leading to muscle atrophy and reduced physical activity (19). A double-blind randomized clinical trial demonstrated that anti-oxidant supplementation improved plasma levels of oxidative stress and inflammation while improving mobility and quality of life in elderly subjects (20). Many studies have also confirmed the ability of EA or SFN to improve oxidative stress and inflammation (21). EA prevents lipopolysaccharide-induced cognitive impairment by inhibiting oxidative stress and neuroinflammation (22). EA reduces the expression of IL-1β, IL-6, and TNF-α in the hippocampus of mice with Alzheimer’s disease (23) and acute myocardial ischemia (24), exerting an anti-inflammatory effect. Other studies have reported that SFN inhibits pro-inflammatory cytokine expression (25). In our study, muscle tissue structure was improved after EA or SFN intervention, collagen/myogenic fibers in skeletal muscle tissue were significantly reduced, and the combined EA+SFN intervention had the most significant effect. In addition, the levels of CAT, SOD1, and SOD2 protein were significantly increased and the levels of inflammatory factors IL-6 and TNF-α were significantly suppressed in the EA and SFN-treated groups compared to SAMP8 mice. These results suggest that EA and SFN can reduce skeletal muscle inflammation and oxidative stress levels in SAMP8 mice, thereby improving skeletal muscles structure and function.

Mitochondrial damage is a marker of reduced muscle function in the elderly and mitochondrial counts are also critical to skeletal muscles function (26). The AMPK/Sirt1/PGC-1α pathway has been shown to improve mitochondrial function (6). In the present experiment, we observed that the muscle tissue of SAMP8 mice had blurred myogenic fibers, deformed and ruptured mitochondria, and a lower number of mitochondria. The expression levels of SIRT1, p-AMPK, and PGC-1α proteins were also low in the muscle tissues of SAMP8 mice. Research shows that promoting phosphorylation of AMPK and activating PGC-1α up-regulated NRF1 expression, enhanced energy metabolism, and inhibited skeletal muscles cell apoptosis (27). In addition, SFN was also found to activate the AMPK/SIRT1/PGC-1ɑ pathway to inhibit apoptosis (28). EA was also able to improve muscle strength by modulating AMPK/PGC-1α signaling in rats with chronic fatigue syndrome by reducing mitochondrial oxidative stress and increasing ATP synthesis (29). A similar phenomenon was observed in our study, where p-AMPK levels were increased, SIRT1 and PGC-1α were activated and skeletal muscle cell apoptosis was inhibited after SFN and EA interventions. At the same time, the number of mitochondria in skeletal muscles was increased and the expression of skeletal muscle atrophy proteins MuRF1 and Atrogin1 was reduced in both SFN and EA intervention groups compared to the SAMP8 group. However, we also found that FOXO3 phosphorylation levels were promoted after p-AMPK activation. Activation of the AMPK/FOXO3 signaling pathway is thought to induce skeletal muscle atrophy in rats (30). Wang *et al*. found that AMPK/FOXO3 signaling could promote mitochondrial dysfunction in an *in vitro* model of skeletal muscles atrophy (31). This suggests the presence of at least two simultaneous pathways regulating skeletal muscle function in sarcopenia following SFN and EA intervention in SAMP8 mice. SFN and EA promote recovery of skeletal muscle function mainly through the p-AMPK/Sirt1/PGC-1α pathway, accompanied by a small activation of the p-AMPK/FOXO3 pathway. Therefore, the study of the optimal intensity and timing of SFN and EA interventions could be the next focus of research. In addition, whether the effects of SFN and EA are reversible is also a question worthy of further exploration.

## Conclusion

In this study, the combined intervention of EA and SFN in SAMP8 mice demonstrated that EA and SFN could inhibit the disease process of sarcopenia in mice by activating the AMPK/Sirt1/PGC-1α pathway. The potential mechanism is through inhibition of skeletal muscles oxidative stress levels, pro-inflammatory factor expression, and skeletal muscle cell apoptosis. The combination of EA and SFN may also provide additional options for sarcopenia prevention and treatment.

## Authors’ Contributions

F G and Z L designed the experiments; F L and Z L performed experiments and collected data; FG discussed the results and strategy; Z L supervised, directed, and managed the study; F G, F L, and Z L approved the final version to be published.

## Funding

The work was supported by the Huzhou Central Hospital Science Foundation for Distinguished Young Scholars (No. 2020YC19). 

## Data Availability Statment

The data that support the findings of this study are available from the corresponding author upon reasonable request.

## Conflicts of Interest

The authors declare that they have no competing interests.
